# Supplementary material for: Chimeric antigen receptor T cells targeting FcRH5 provide robust tumour-specific responses in murine xenograft models of multiple myeloma
Source: Nat Commun. 2023 Jun 20;14:3642. doi: 10.1038/s41467-023-39395-4 (PMC10282100; doi:10.1038/s41467-023-39395-4)
Supplement: Supplementary file 1 — Supplementary Information [file 41467_2023_39395_MOESM1_ESM.pdf]

# Supplementary Information

## Chimeric antigen receptor T cells targeting FcRH5 provide robust tumour-specific responses in murine xenograft models of multiple myeloma

Dongpeng Jiang[1], Haiwen Huang[2], Huimin Qin[1], Koukou Tang[1], Xiangru Shi[1], Tingting Zhu[1], Yuqing Gao[1], Ying Zhang[1], Xiaopeng Tian[2], Jianhong Fu[2], Weiwei Qu[1], Weilan Cai[1], Yang Xu[1*], Depei Wu[1*], Jianhong Chu[1*]

[1] Institute of Blood and Marrow Transplantation, National Clinical Research Center for Hematologic Diseases, Jiangsu Institute of Hematology, The First Affiliated Hospital of Soochow University, Collaborative Innovation Center of Hematology, Soochow University, Suzhou, Jiangsu, China

[2] Department of hematology, The First Affiliated Hospital of Soochow University, Suzhou, Jiangsu, China

Supplementary figures and legends 1 to 15

Supplementary table 1

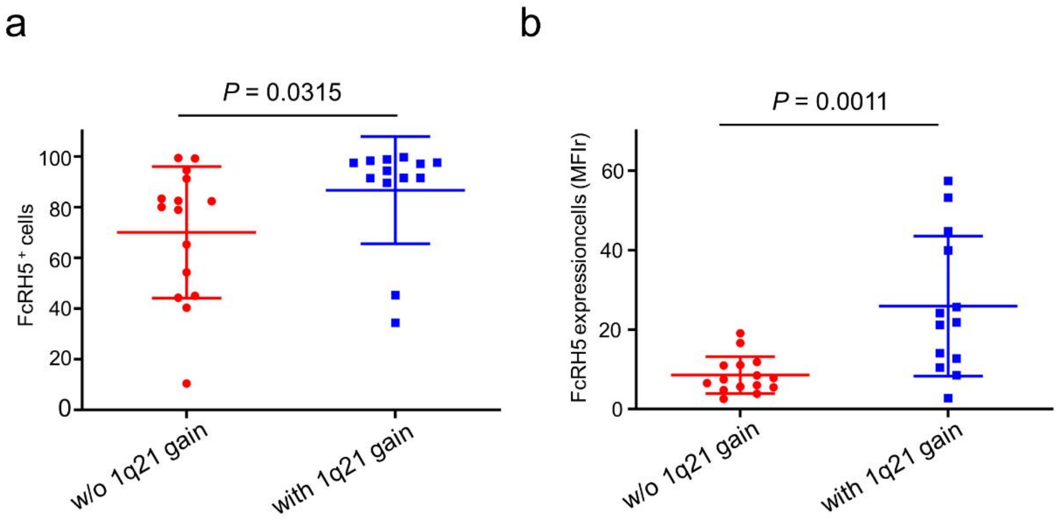


**Supplementary Figure 1. Flow cytometry analysis of FcRH5 protein expression levels in bone marrow biopsies from MM patients with or without (w/o) 1q21 gain.** (a) Graph depicted the percentage of FcRH5 positive cells among $CD138^+$ myeloma cells. Mean±SD, unpaired two-tailed t-test. n=15 for those w/o 1q21 gain, n=13 for MM patients with 1q21 gain. (b) Graph depicted the MFIr of FcRH5 protein on myeloma cells. Mean±SD, unpaired two-tailed t-test. n=15 for those w/o 1q21 gain, n=13 for MM patients with 1q21 gain.

a

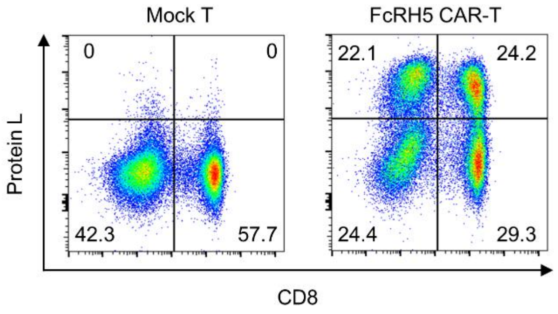


b

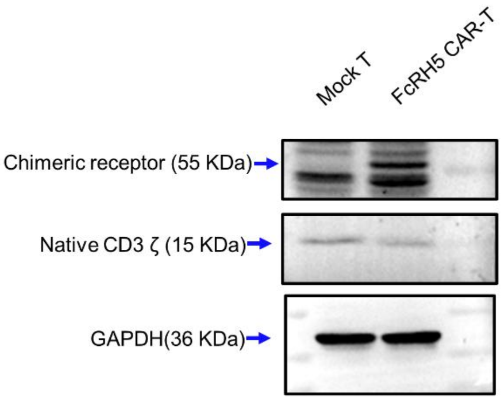


**Supplementary Figure 2. Validation of CAR expression on the surface of FcRH5 CAR-T cells.**

(a) Mock T and FcRH5 CAR-T cells were labeled with biotin-protein L followed by staining with APC-streptavidin and BV421-conjugated anti-CD8 antibody. The numbers on the plots were the percentages of cells in each quadrant. (b) The protein lysates from mock T cells and FcRH5 CAR-T cells were probed with anti-CD3ζ primary antibody. Experiment was repeated with 2 different T cell donors.

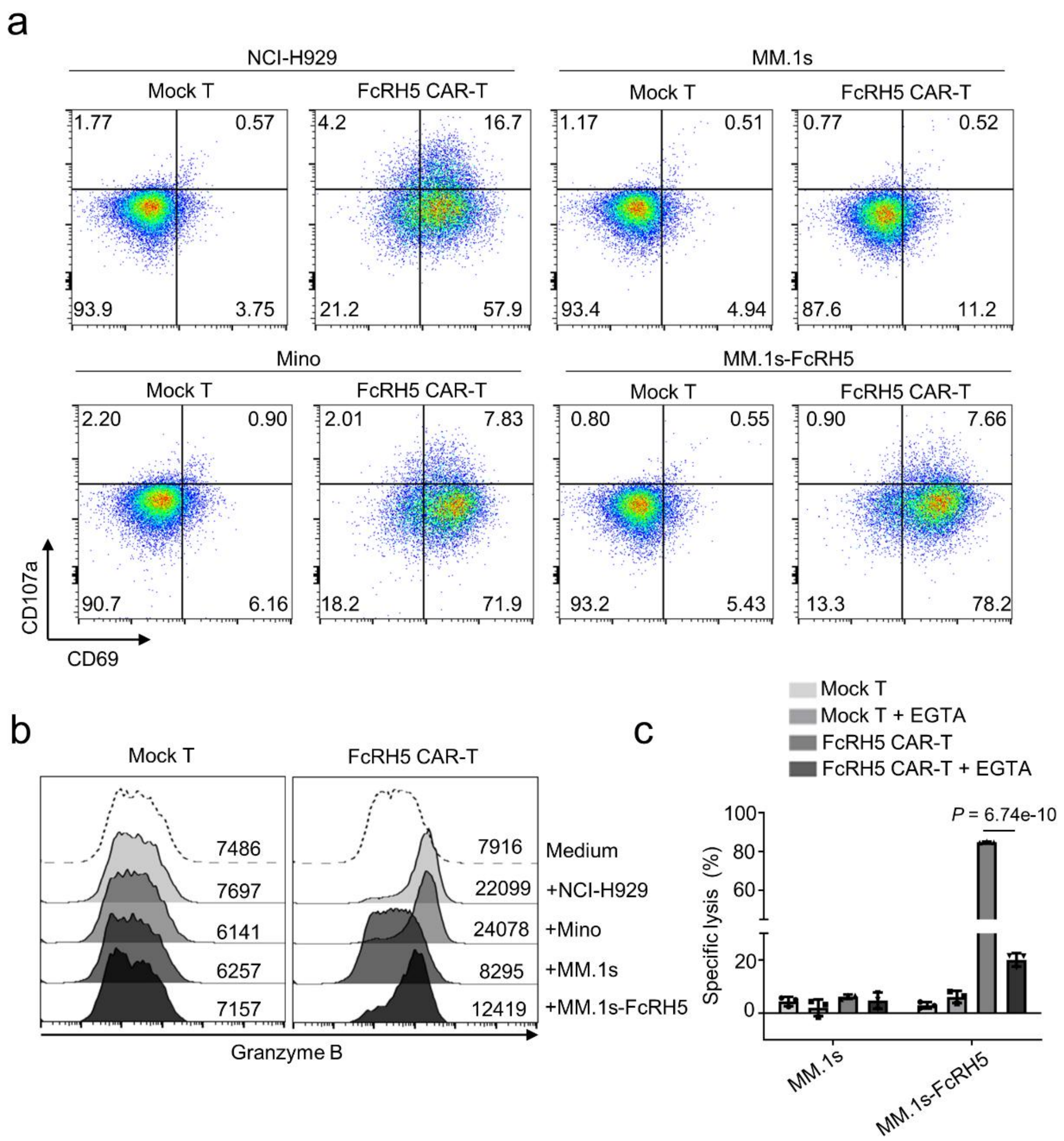


**Supplementary Figure 3. FcRH5 CAR-T cells expresses activation markers, form immune synapse in response to target cells and lyse target cells in a granzyme B-dependent manner.**

(a) Expression of the CD107a and CD69 on the surface of mock T or FcRH5 CAR-T cells was evaluated by flow cytometry following 4 hours of co-culture with indicated target cells. (b) Granzyme B expression on mock T or FcRH5 CAR-T cells was determined by flow cytometric analysis in response to different target cells. The numbers marked represent the mean fluorescence intensity (MFI). (c) The cytolytic effect of mock T or FcRH5 CAR-T cells was determined after co-cultured with target cells in the presence or absence of 10 nM EGTA capable of inhibiting granzyme B release. Mean±SD, n=3 biologically independent co-cultures, one-way ANOVA with *post hoc* Tukey-Kramer test. (FcRH5 CAR-T v.s. FcRH5 CAR-T+EGTA). Data shown are representative for results obtained in independent experiments with T cells from three donors.

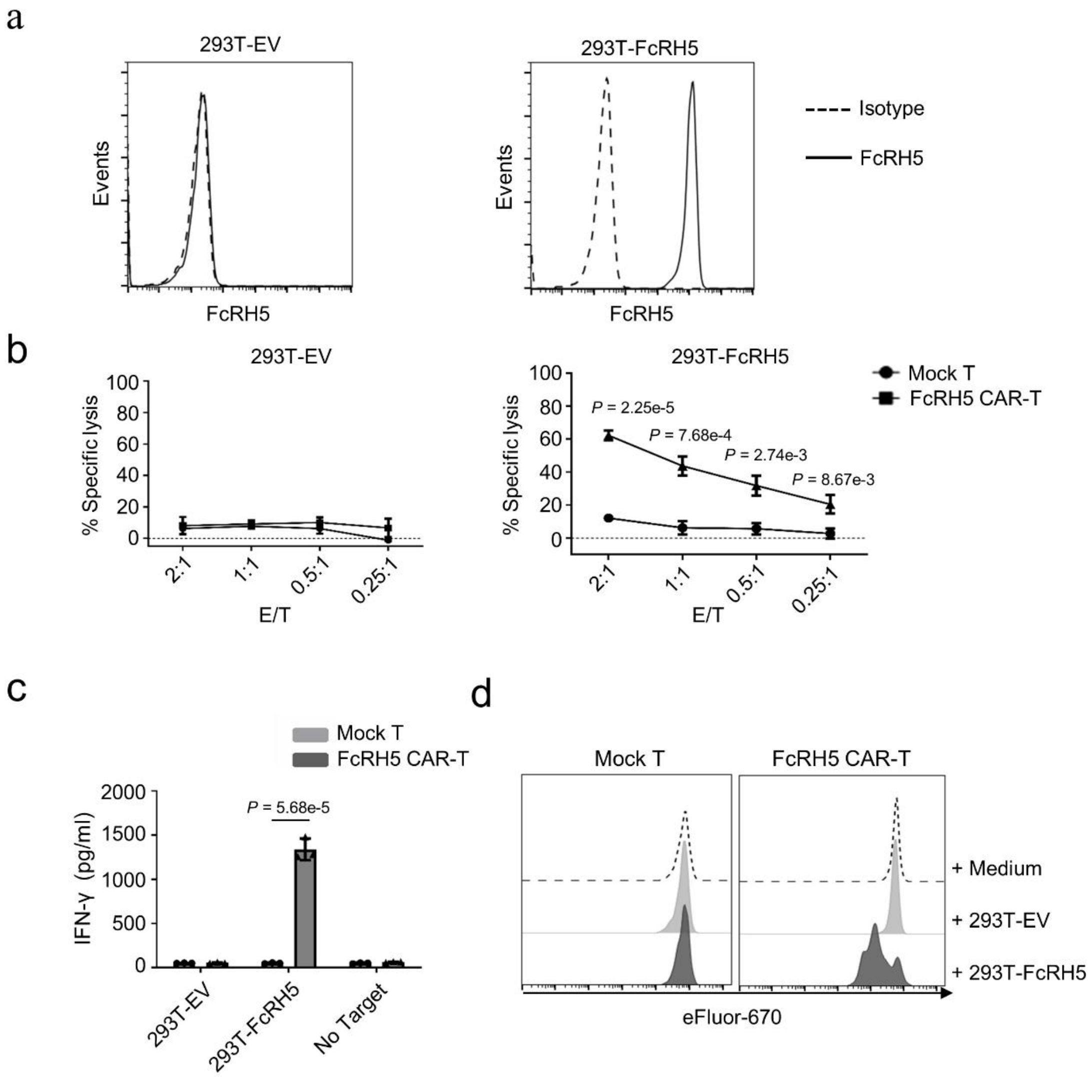


**Supplementary Figure 4. Forced expression of FcRH5 on the surface of 293T cells confers the susceptibility to FcRH5 CAR-T cells.** (a) 293T cells were transduced with lentiviruses encoding human FcRH5 or empty vector (EV), and the surface expression of FcRH5 was confirmed by flow cytometry analysis. (b) 293T-EV or 293T-FcRH5 cells previously transduced with click beetle red (CBR) luciferase were co-cultured with mock T or FcRH5 CAR-T cells for 6 hours at indicated effector: target cell (E/T) ratios, and the cytolytic effect was determined by luciferase-based cytotoxicity assay. Mean±SD, n=3 biologically independent co-cultures, two-tailed Student's t-test. (c) Mock T or FcRH5 CAR-T cells were co-cultured with 293T-EV or 293T-FcRH5 cells for 24 hours, and the cell-free supernatants were harvested and assessed for IFN-γ release. Mean±SD, n=3 biologically independent co-cultures, two-tailed Student's t-test. (d)

293T-EV or 293T-FcRH5 cells pre-labeled with eFlour-670 were co-cultured with equal number of mock T or FcRH5 CAR-T cells for 5 days, and cell division was determined. The plots were gated on $CD3^{+}$ T cells. The above experiments b-d were repeated with 2 different T cell donors.

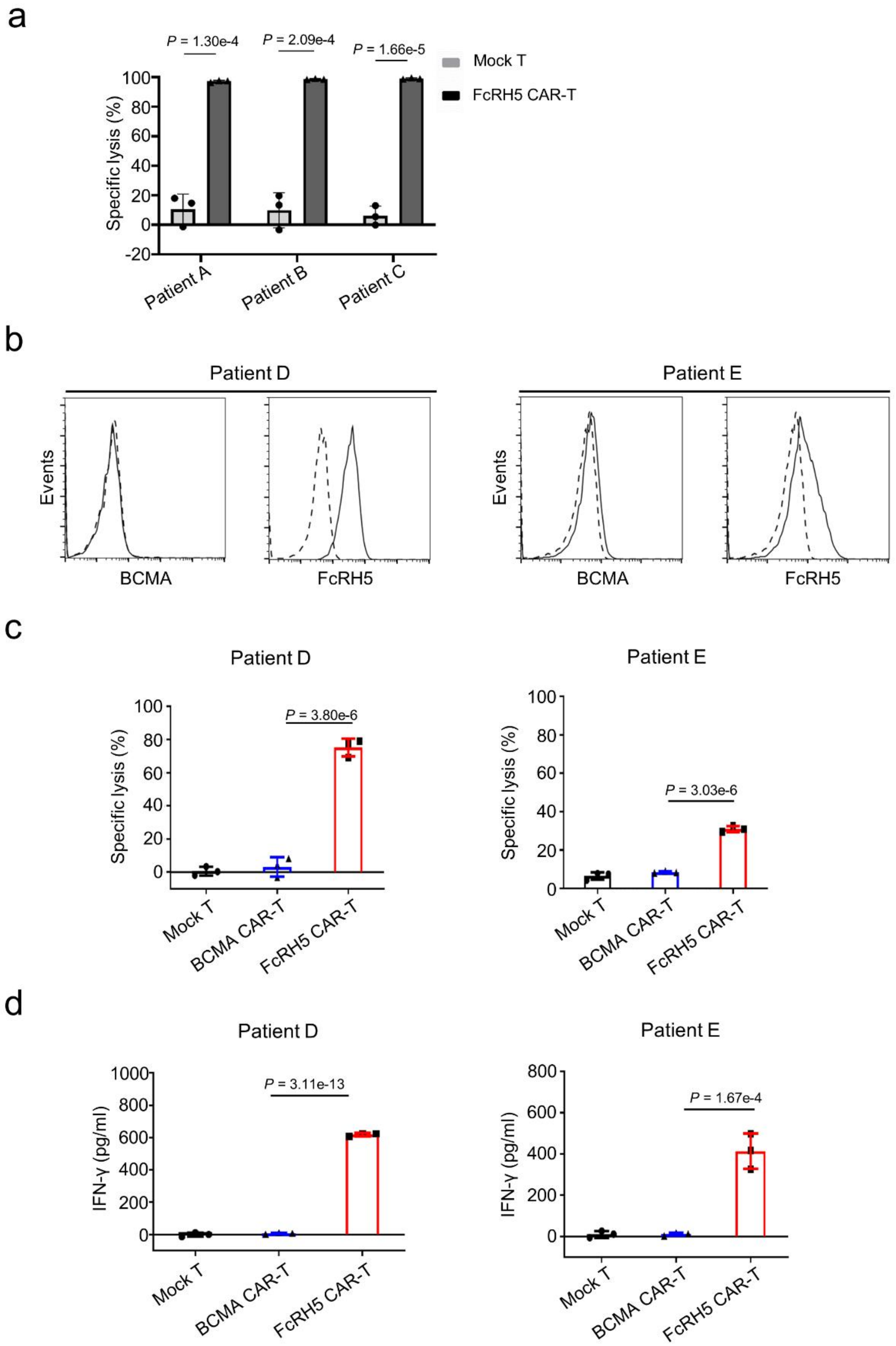


**Supplementary Figure 5. Primary myeloma cells from two MM patients lacking BCMA expression can be recognized and killed by autologous FcRH5 CAR-T cells.** (a) Related to Fig.2e,g. The indicated patient-derived primary myeloma cells were labeled with eFlour-670 and co-cultured with autologous mock T or FcRH5 CAR-T cells at the E/T ratio of 5:1 for 24 hours,

and then cytolytic effect of T cells was determined by a flow cytometry-based cytotoxicity assay. Mean±SD, n=3 biologically independent co-cultures, unpaired two-tailed Student t test. (b) Flow cytometric analysis of BCMA and FcRH5 expression on $CD138^+$ primary myeloma cells from a newly-diagnosed patient (Patient D) and a relapsed MM patient (Patient E). Solid line represents staining with anti-FcRH5 or anti-BCMA mAb and dashed line represents staining with isotype control antibody. (c) The $CD138^+$ primary myeloma cells were labeled with eFlour-670 and co-cultured with autologous mock T, BCMA CAR-T or FcRH5 CAR-T cells at the E/T ratio of 5:1 for 6 hours, and the cytolytic effect was determined by a flow cytometry-based assay. Mean±SD, n=3 biologically independent co-cultures, one-way ANOVA with *post hoc* Tukey-Kramer test for multiple comparisons. (d) The primary myeloma cells were co-cultured with autologous mock T, BCMA CAR-T or FcRH5 CAR-T cells for 24 hours, and cell-free supernatants were harvested for determination of IFN-γ secretion. Mean±SD, n=3 biologically independent co-cultures, one-way ANOVA with *post hoc* Tukey-Kramer test for multiple comparisons.

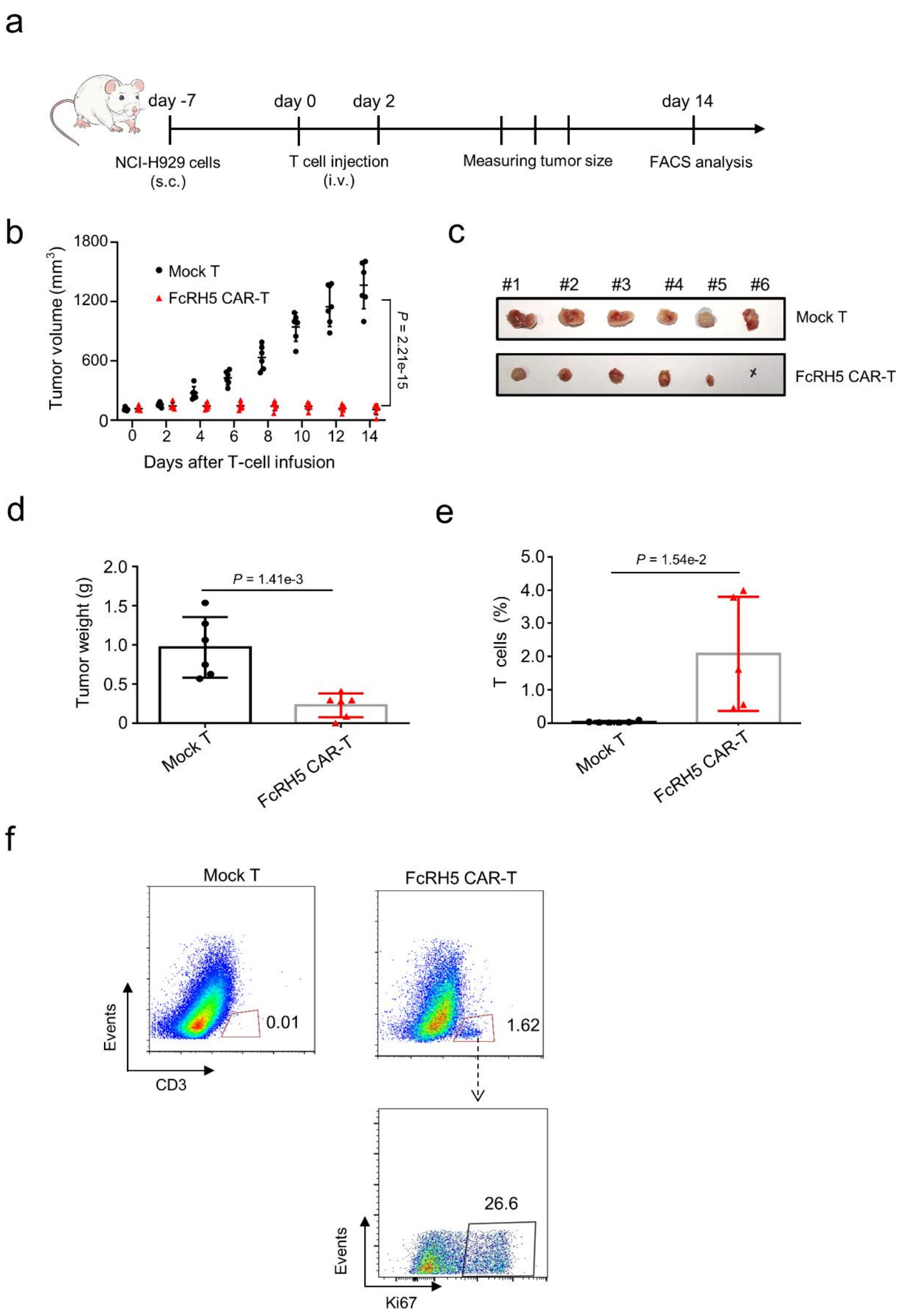
a
day -7
day 0
day 2
day 14
NCI-H929 cells (s.c.)
T cell injection (i.v.)
Measuring tumor size
FACS analysis
b
Tumor volume (mm3)
Mock T
FcRH5 CAR-T
P = 2.21e-15
Days after T-cell infusion
c
#1 #2 #3 #4 #5 #6
Mock T
FcRH5 CAR-T
d
Tumor weight (g)
P = 1.41e-3
Mock T
FcRH5 CAR-T
e
T cells (%)
P = 1.54e-2
Mock T
FcRH5 CAR-T
f
Mock T
FcRH5 CAR-T
0.01
1.62
Events
CD3
26.6
Events
Ki67

**Supplementary Figure 6. FcRH5 CAR-T cells display potent tumoricidal activity in the subcutaneous NCI-H929 cell tumor xenograft model.** (a) Experimental design. Male NOG mice aged 6-8 weeks were subcutaneously inoculated with $5x10^6$ NCI-H929 cells on day -7, and were then intravenously infused with $5x10^6$ mock T or FcRH5 CAR-T cells (n=6 mice per group) on day 0 and day 2. (b) Graph showed the change in the tumor volume at different time points with the lines connecting means. Mean±SD, n=6 mice, two-way ANOVA with Šídák's correction for multiple comparisons. (c) Images of tumor nodules harvested from different treatment groups on day 14, and "x" indicated no tumor available in the corresponding mouse. (d) Tumor weight was quantified in each treatment group at the time of sacrifice. Mean±SD, n=6 mice, unpaired two-tailed Student t test, (e) The percentage of T cells (gated on human $CD3^+$) within tumor nodules was determined by flow cytometric analysis. Mean±SD, n=6 mice for Mock T group and n=5 mice for FcRH5 CAR-T group, unpaired two-tailed Student t test, (f) Representative result of Ki67 staining indicated that the tumor-infiltrating FcRH5 CAR-T cells retained the expansive capacity.

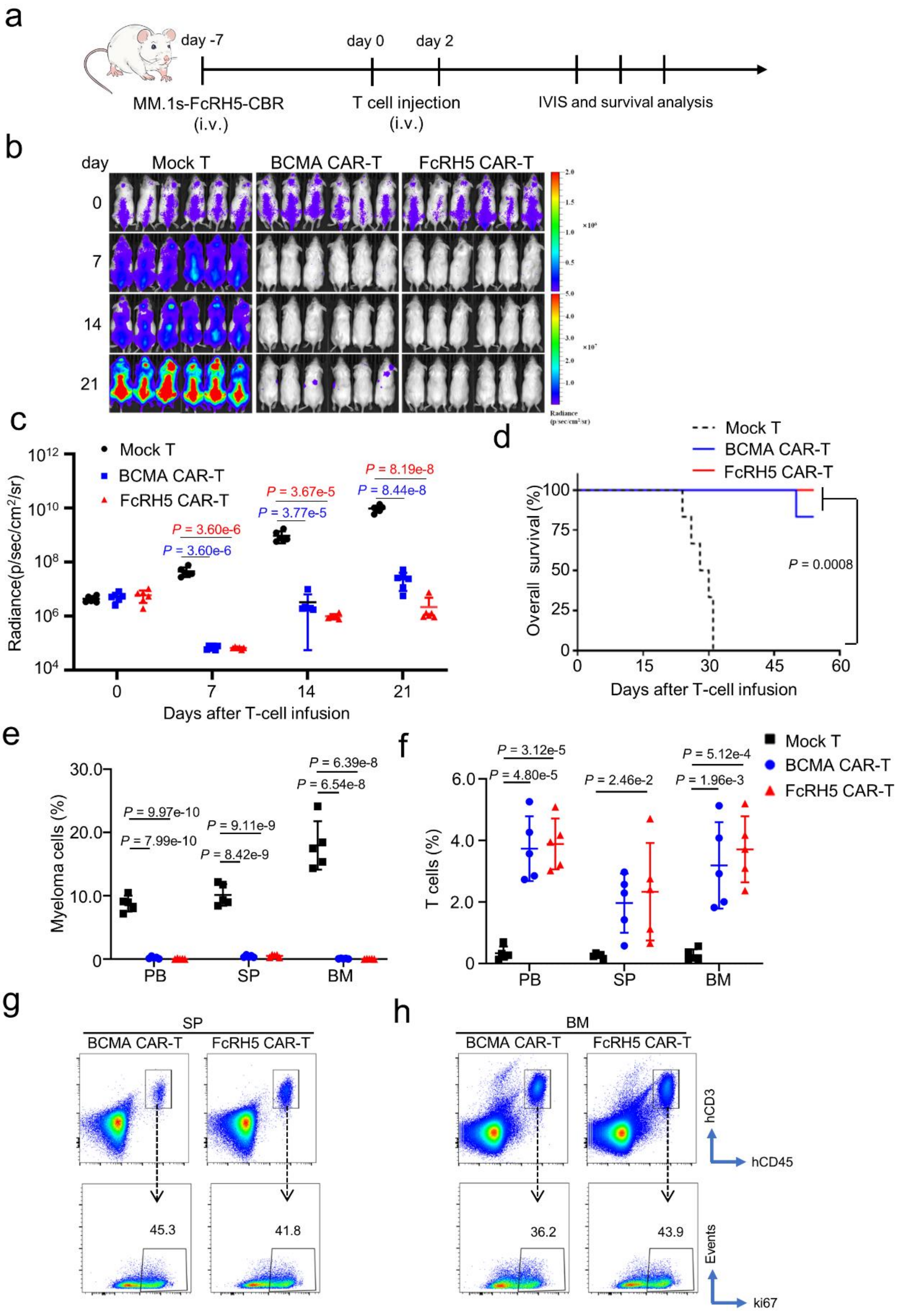


**Supplementary Figure 7. FcRH5 CAR-T cells induce durable tumor regression in a disseminated MM xenograft model.** (a) Experimental schematic: Male NOG mice aged 6-8 weeks were intravenously inoculated with 5x$10^6$ MM.1s-FcRH5-CBR cells on day -7, and were then treated with 5x$10^6$ mock T, FcRH5 CAR-T cells or BCMA CAR-T cells (n=6 mice per group)

on day 0 and day 2. (b) Serial bioluminescence (BL) imaging to assess tumor burden for each group at different time points. Note the scale indicating upper and lower BL thresholds at each time point. (c) Quantification of whole-body luminescence in NOG mice from each group at different time points with the lines connecting means. Mean±SD, n=6 mice, unpaired two-tailed Student t test, One-way ANOVA with Dunnett's correction for multiple comparison. (d) Kaplan-Meier curve for the overall survival of mice from different treatment group. n=6 mice per group, Log-rank (Mantel-Cox) test. (d-g) In a parallel experiment with the same tumor inoculation and treatment regimen (n=5 mice per group), the mice were sacrificed three weeks after T cell inoculation, and analyzed for the presence of myeloma cells and T cells in peripheral blood (PB), spine (SP) and bone marrow (BM). (e) Flow cytometric analysis of the frequency of myeloma cells (gated on human $CD138^{+}$) in PB, SP and BM. Graph displays individual and mean values. Mean±SD, n=5 mice, One-way ANOVA with Dunnett's correction for multiple comparison. (f) Flow cytometric analysis of the frequency of T cells (gated on human $CD45^{+}CD3^{+}$) in PB, SP and BM. Graph displays individual and mean values. Mean±SD, n=5 mice, One-way ANOVA with Dunnett's correction for multiple comparison. (g) Representative flow cytometric analysis of Ki67 expression on T cells (human $CD45^{+}CD3^{+}$) from the spine of mice treated with indicated CAR-T cells. (h) Representative flow cytometric analysis of Ki67 expression on T cells (human $CD45^{+}CD3^{+}$) from the bone marrow of mice treated with indicated CAR-T cells.

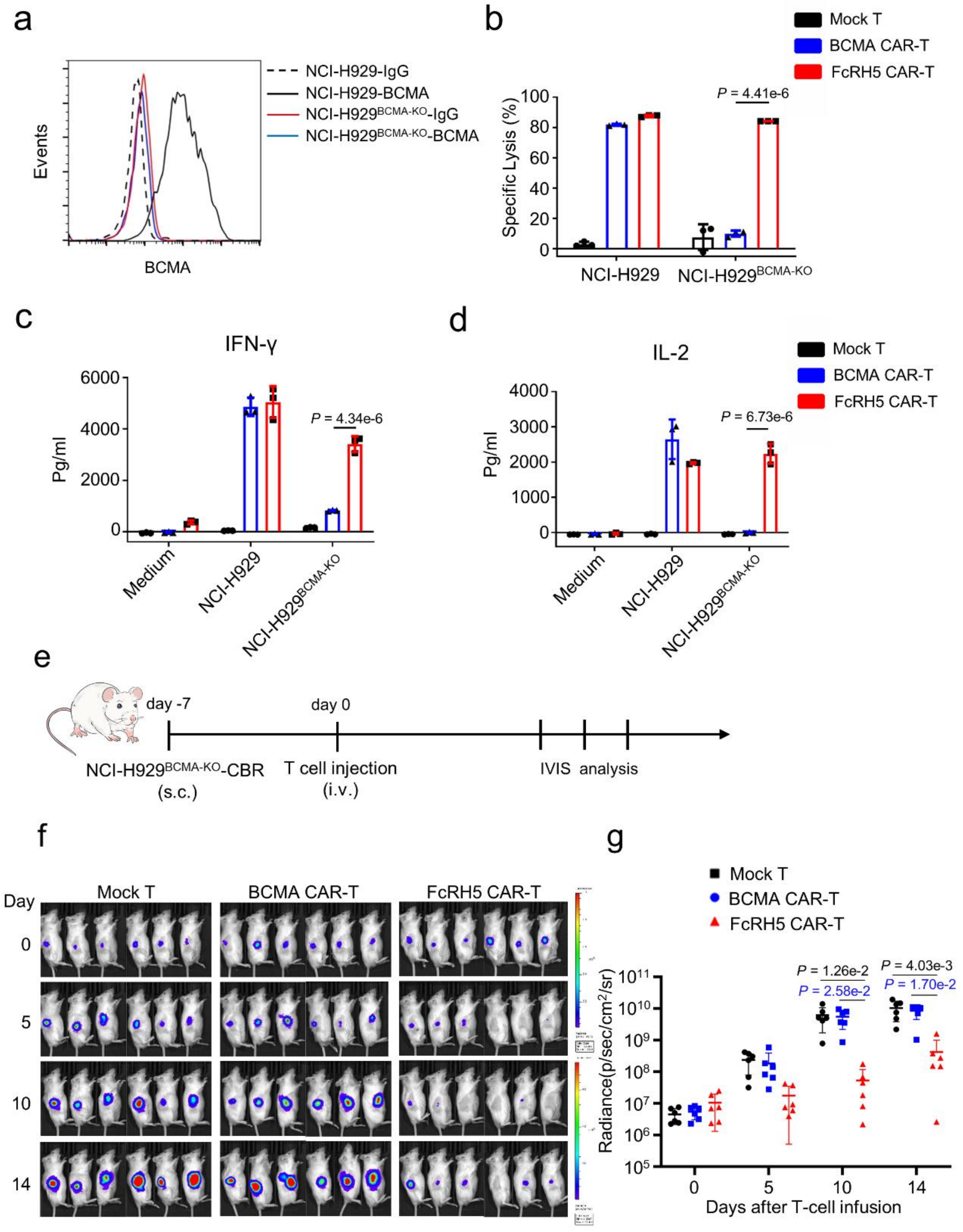


**Supplementary Figure 8. FcRH5 CAR-T cells recognize and eradicate NCI-H929 cells with BCMA knockout both *in vitro* and *in vivo*.** (a) Flow cytometric analysis of BCMA expression on the surface of NCI-H929 and NCI-H929$^{BCMA-KO}$ cells. (b) H929 or H929$^{BCMA-KO}$ cells previously transduced with click beetle red (CBR) luciferase were co-cultured with mock T, BCMA CAR-T or FcRH5 CAR-T cells for 24 hours at the effector: target cell (E/T) ratio of 1:1, and the cytolytic

effect was determined by luciferase-based cytotoxicity assay. Mean±SD, n=3 biologically independent co-cultures, one-way ANOVA with *post hoc* Tukey-Kramer test. (c,d) Mock T, BCMA CAR-T or FcRH5 CAR-T cells were co-cultured with equal number of NCI-H929 or NCI-H929$^{BCMA-KO}$ cells for 24 hours, and the cell-free supernatants were harvested and assessed for IFN-γ (c) and IL-2 (d) release, respectively. Mean±SD, n=3 biologically independent co-cultures, one-way ANOVA with *post hoc* Tukey-Kramer test. The above experiment b-d was repeated with 2 different T cell donors. (e) Experimental schematic: Male NOG mice aged 6-8 weeks were subcutaneously inoculated with $5x10^6$ NCI-H929$^{BCMA-KO}$-CBR cells on day -7, and were then intravenously infused with $5x10^6$ mock T, FcRH5 CAR-T, BCMA CAR-T cells (n=6 mice per group for each donor) from two different donors when the tumors became palpable (day 0). (f) Representative tumor bioluminescence of NOG mice at different time points. (g) Graph showed the quantification of whole body luminescence in NOG mice from each group at different time points with the lines connecting the mean values. Data shown are representative for results obtained in independent experiments with T-cell from 2 donors. Mean±SD, n=6 per group. One-way ANOVA followed by Bonferroni correction for multiple comparisons (two-sided).

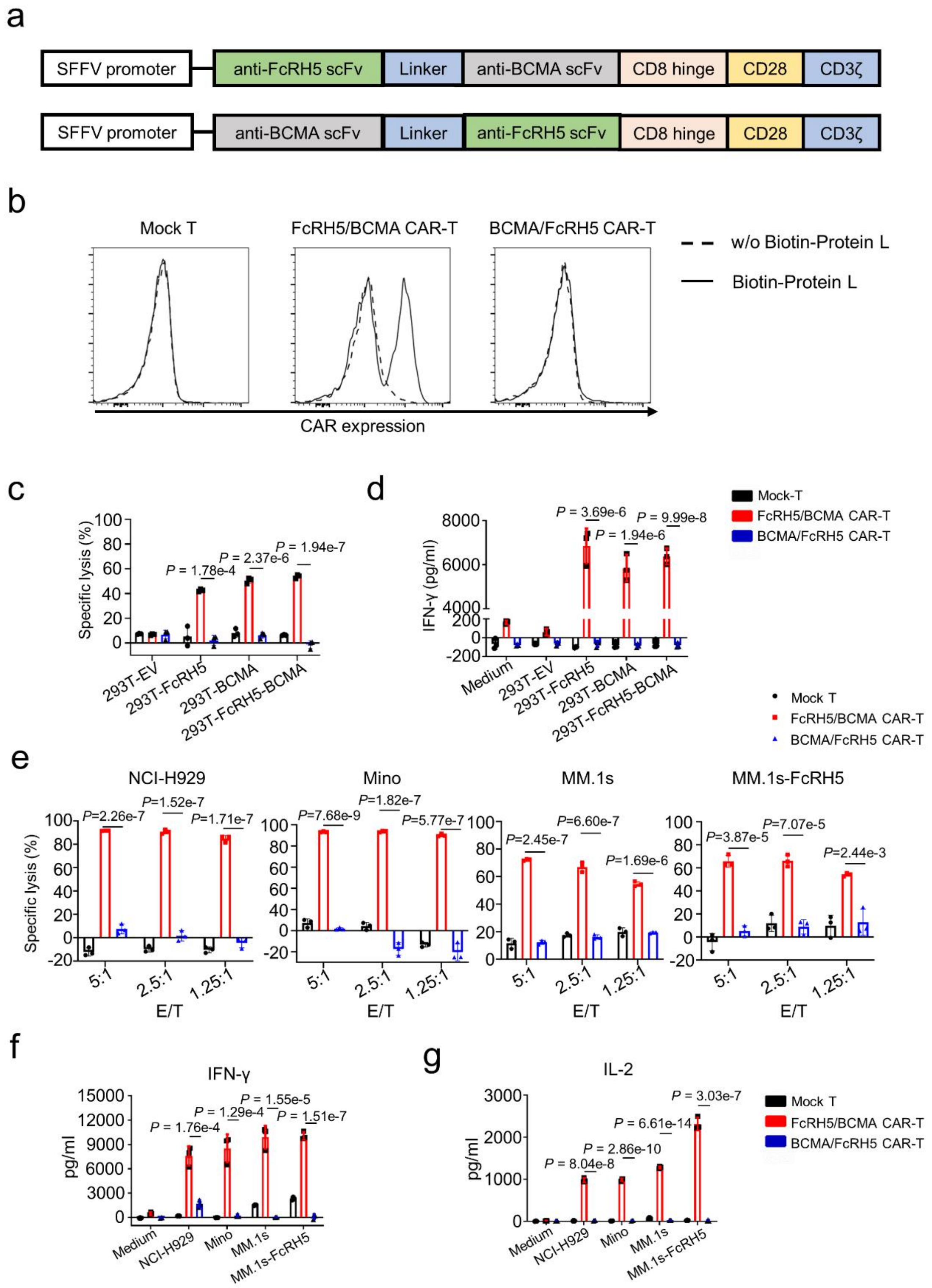


**Supplementary Figure 9. Comparison of two forms of bispecific CAR-T cells.** (a) Two second generation bispecific CARs incorporating CD28 costimulatory moiety were constructed with different orientation of the scFvs in tandem, with FcRH5/BCMA CAR on the top, BCMA/FcRH5 CAR on the bottom. (b) Mock T, FcRH5/BCMA CAR-T or BCMA/FcRH5 CAR-T cells were

stained with biotin-labeled Protein L followed by incubation with APC-streptavidin, and the CAR expression was analyzed by flow cytometry. (c) Specific lysis of 293T cells overexpressing FcRH5 and BCMA alone or in combination by bispecific CAR-T cells was determined by the 6-hour luciferase-based cytolytic assay. Mean±SD, n=3 biologically independent co-cultures, unpaired two-tailed Student *t* test. (d) IFN-γ release by bispecific CAR-T cells in response to 293T cells overexpressing FcRH5 and BCMA alone or in combination was determined by ELISA. Mean±SD, n=3 biologically independent co-cultures, unpaired two-tailed Student *t* test. (e) The cytolytic activity of mock T, FcRH5/BCMA CAR-T or BCMA/FcRH5 CAR-T cells was determined after co-cultured with indicated target cells for 6 hours. Mean±SD, n=3 biologically independent co-cultures. (f) IFN-γ release by mock T, FcRH5/BCMA CAR-T or BCMA/FcRH5 CAR-T cells after co-cultured with indicated target cells for 24 hours was assessed by ELISA. Mean±SD, n=3 biologically independent co-cultures, one-way ANOVA with *post hoc* Tukey-Kramer test. (g) IL-2 secretion by mock T, FcRH5/BCMA CAR-T or BCMA/FcRH5 CAR-T cells following co-culture with indicated target cells for 24 hours was evaluated by ELISA. Mean±SD, n=3 biologically independent co-cultures, One-way ANOVA with *post hoc* Tukey-Kramer test. The above experiments b-g were repeated with 2 different T cell donors.

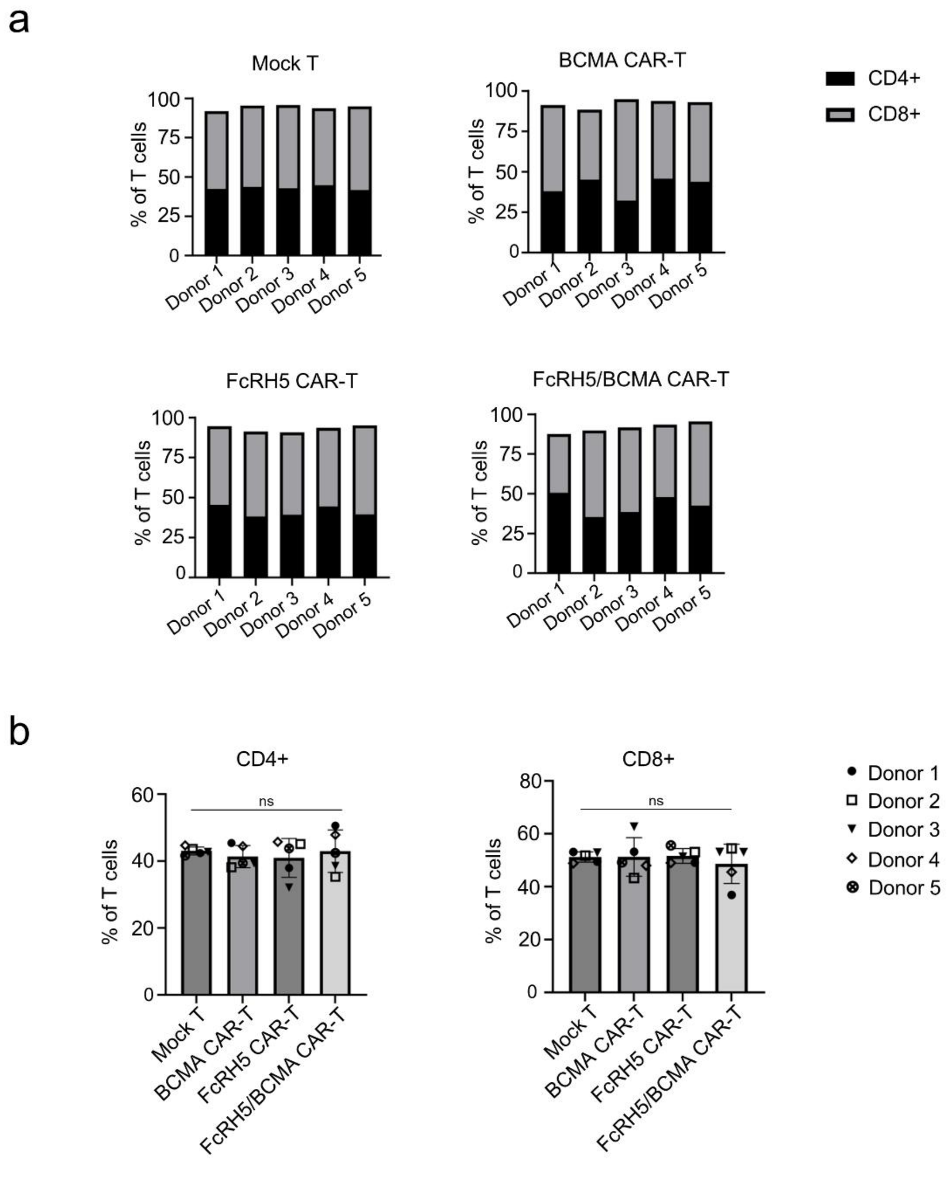


**Supplementary Figure 10. CD4/CD8 composition of CAR T-cells.** (a) Bar graphs showing the frequency of CD4 positive and CD8 positive mock T, monospecific CAR-T and bispecific CAR T-cells from the indicated donors. (b) Statistical analysis denoting no significant difference in the proportion of CD4 and CD8 positive cells among CAR-T cells from different donors. One-way ANOVA followed by Bonferroni correction for multiple comparisons (two-sided). Mean±SD, n=5 biologically independent experiments.

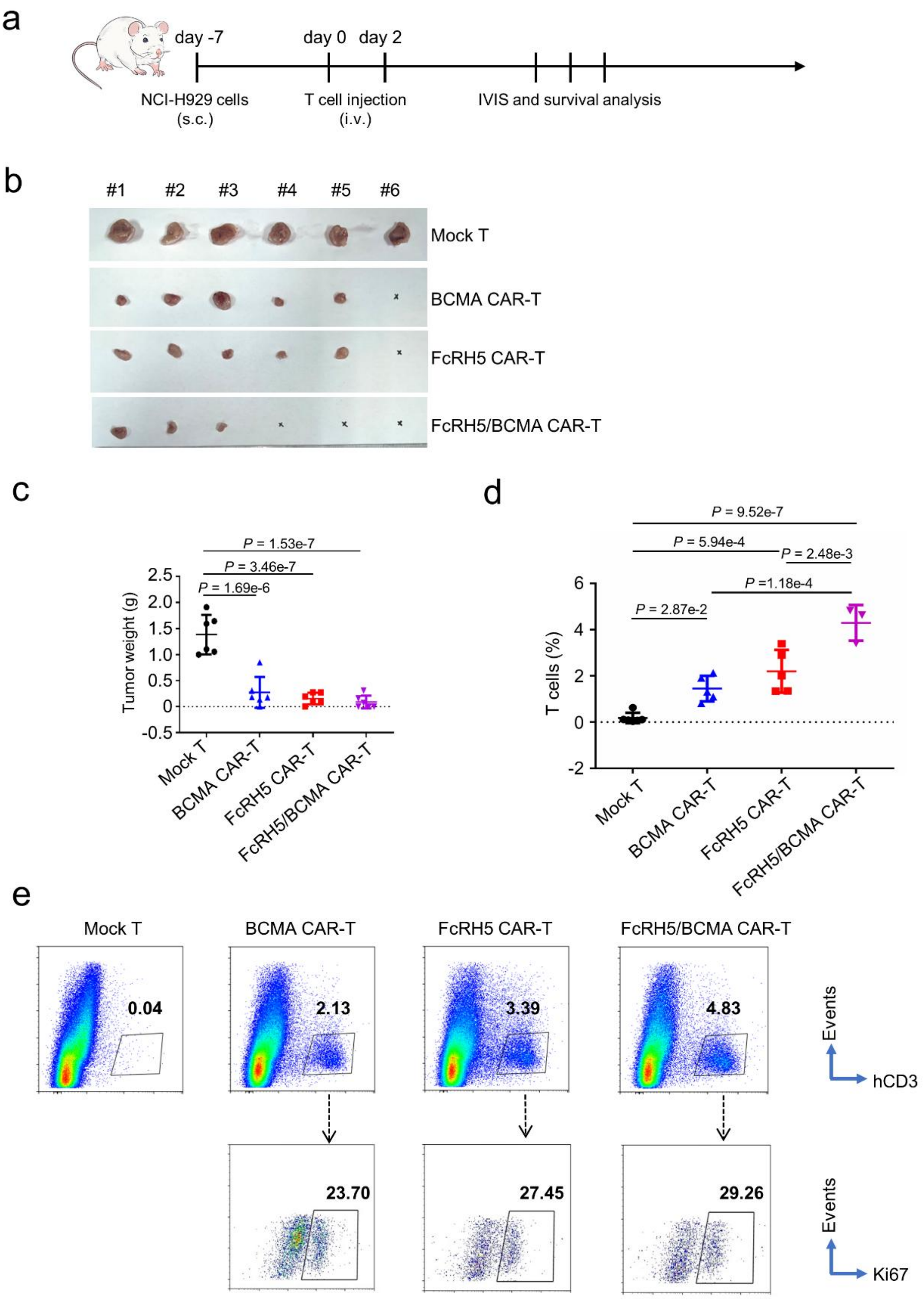


**Supplementary Figure 11. FcRH5/BCMA bispecific CAR-T cells displayed improved tumoral infiltration than mono-specific CAR-T cells.** (a) Experimental schematic: Male NOG mice aged 6-8 weeks were subcutaneously inoculated with $5x10^6$ NCI-H929 cells on day -7, and

were then intravenously infused with $5x10^6$ mock T, FcRH5 CAR-T, BCMA CAR-T or FcRH5/BCMA CAR-T cells (n=6 mice per group) on day 0 and day 2. On day 14, mice were sacrificed, and tumors were dissected, weighed and subjected to flow cytometry analysis. (b) Graph showed the images of tumor nodules harvested from indicated treatment groups. (c) Graph shows quantitative analysis of the tumor weight in each group. Mean ± SD, n=6, one-way ANOVA with *post hoc* Tukey-Kramer test. (d) The percentage of human $CD3^+$ T cells within the dissected tumors was quantified by flow cytometry. Mean±SD, n=6 mice for mock T group, n=5 mice for BCMA CAR-T and FcRH5 group, n=3 for FcRH5/BCMA CAR-T group, one-way ANOVA with Bonferroni's correction for multiple comparison. Mean ± SD. (e) Representative flow cytometric analysis of Ki67 expression on the indicated tumor-infiltrating mono-specific or bispecific CAR-T cells from each group.

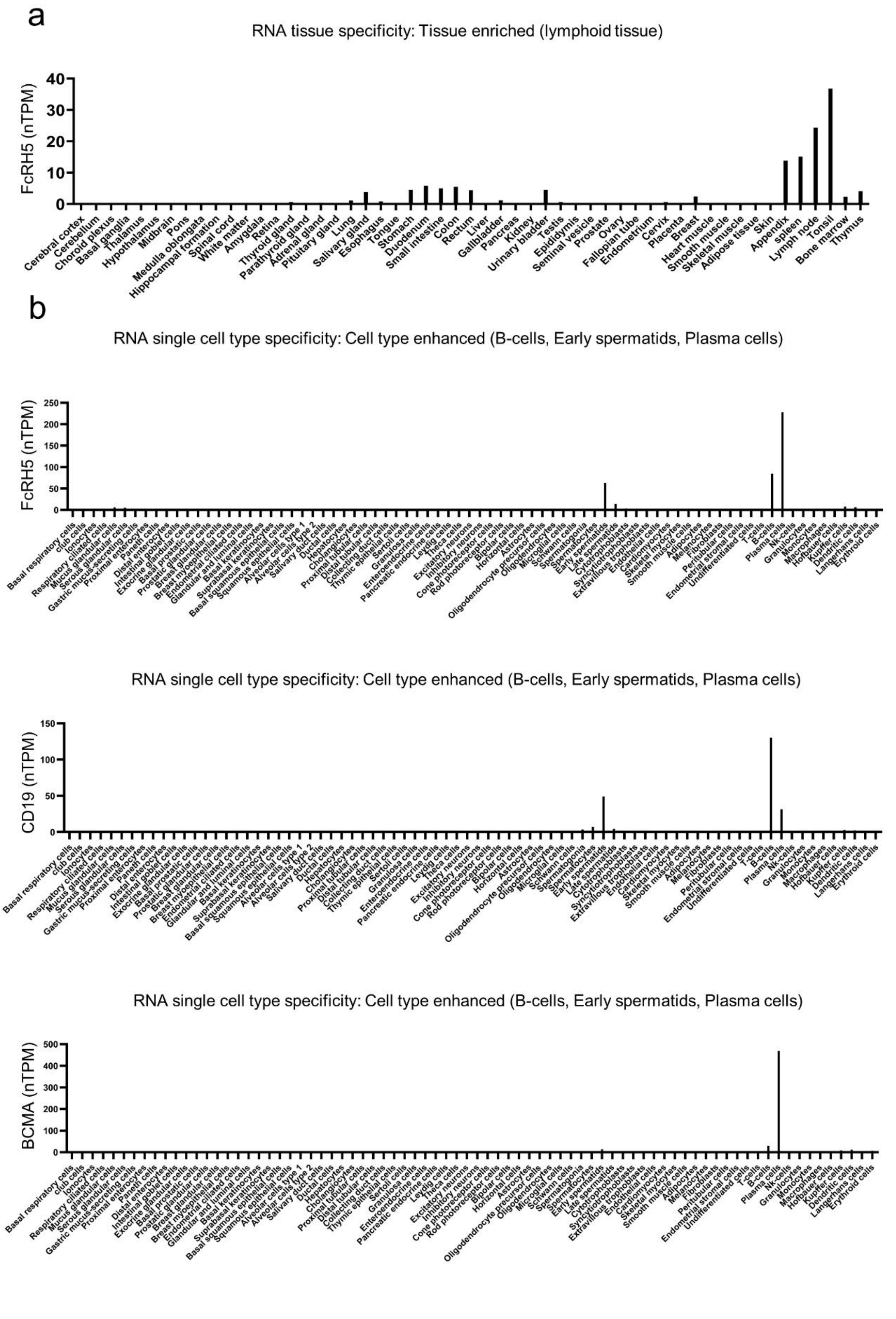


**Supplementary Figure 12. Analysis of FcRH5 transcript expression in the Human Protein Atlas.** (a) The Consensus transcript expression levels for FcRH5 were summarized in 54 human tissues based on transcriptomics data from the two sources HPA and GTEx datasets

(https://www.proteinatlas.org/ENSG00000143297-FCRL5/tissue.). The consensus nTPM value for each gene and tissue type represents the maximum nTPM value based on HPA and GTEx. (b) Single cell RNA sequencing (scRNAseq) data of FcRH5, CD19 and BCMA from 29 human tissues and peripheral blood mononuclear cells (PBMCs) retrieved from the Single Cell Expression Atlas, the Human Cell Atlas, the Gene Expression Omnibus, the Allen Brain Map, European Genome-phenome Archive and the Tabula Sapiens. (https://www.proteinatlas.org/ENSG00000143297-FCRL5/single+cell+type; https://www.proteinatlas.org/ENSG00000177455-CD19/single+cell+type; https://www.proteinatlas.org/ENSG00000048462-TNFRSF17/single+cell+type)

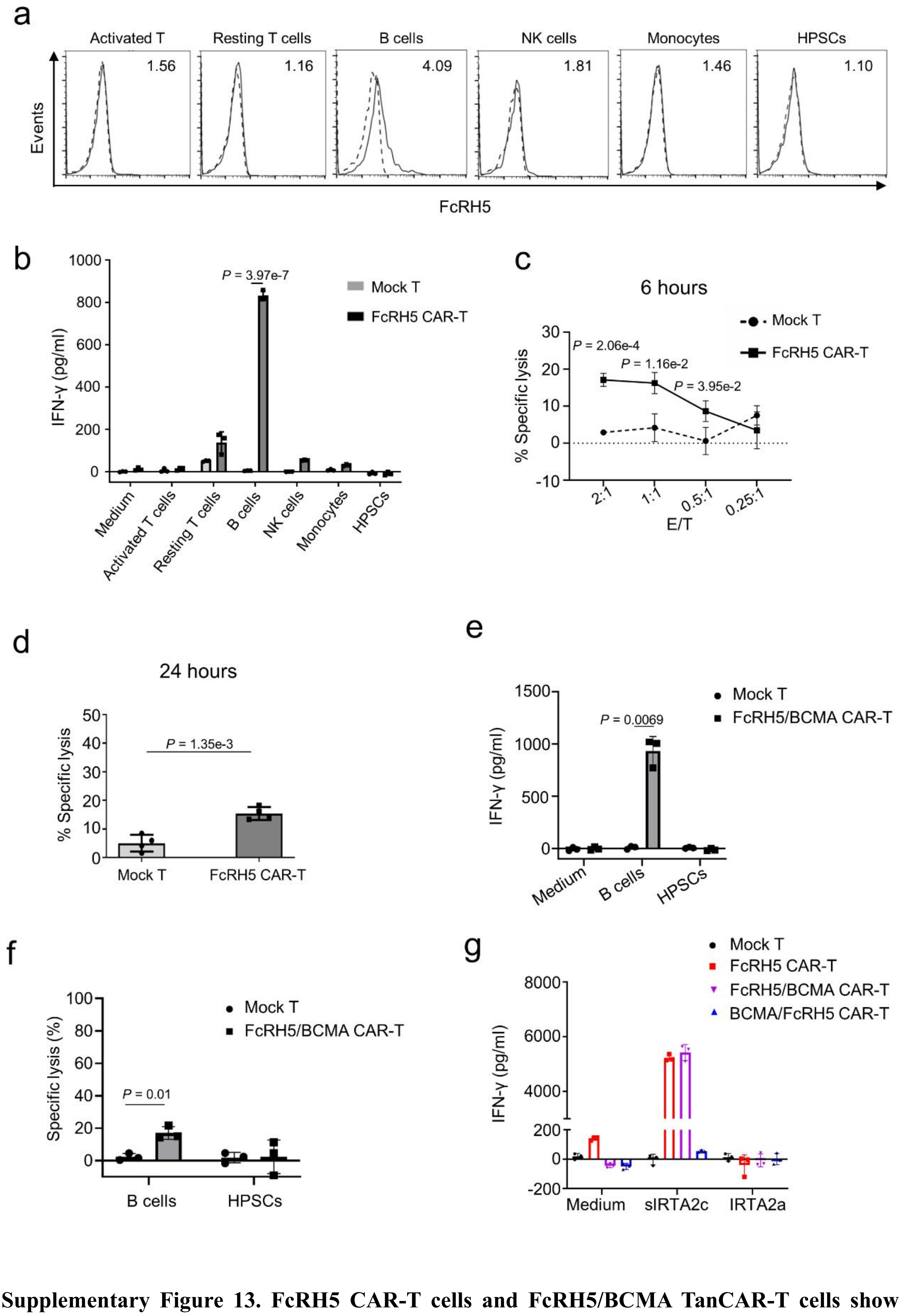


**Supplementary Figure 13. FcRH5 CAR-T cells and FcRH5/BCMA TanCAR-T cells show minimal reactivity with normal B cells.** (a) Resting T cells ($CD56^-CD3^+$), OKT3-activated T cells ($CD56^-CD3^+$), B cells ($CD19^+$), NK cells ($CD56^+CD3^-$), monocytes ($CD14^+$) and $CD34^+$ hematopoietic progenitor/stem cells (HPSCs) were stained with APC-conjugated anti-FcRH5

antibody followed by flow cytometry analysis to evaluate surface expression of FcRH5. Inset numbers denote the MFIr. The experiment was repeated with 4 different donors. (b) The indicated subsets derived from one donor were co-cultured with mock T or FcRH5 CAR-T cells for 24 hours, and cell-free supernatants were harvested for assessment of IFN-γ secretion. Mean±SD, n=3 biologically independent co-cultures, two-tailed Student *t*-test.. Experiment was repeated with 3 different donors. (c) The 6-hour cytotoxicity of mock T or FcRH5 CAR-T cells against $CD19^+$ B cells from one normal donor at the indicated E/T ratios was determined. Mean±SD, n=3 biologically independent co-cultures. Experiment was repeated with 2 different donors. (d) The 24-hour cytotoxicity of mock T or FcRH5 CAR-T cells against $CD19^+$ B cells isolated from four different normal donors at the E/T ratio of 2:1 was determined. Mean±SD, n=4, two-tailed Student *t*-test. (e) Mock T or FcRH5/BCMA TanCAR-T cells were co-cultured with B cells and HPSCs for 24 hours, and cell-free supernatants were collected for assessment of IFN-γ secretion. Mean±SD, n=3 biologically independent co-cultures, two-tailed Student *t*-test. Experiment was repeated with 2 different donors. (f) The 24-hour cytotoxicity of mock T or FcRH5/BCMA TanCAR-T cells against B cells and HPSCs from three different donors at the E/T ratio of 2:1 was determined. Mean±SD, n=3, two-tailed Student *t*-test. (g) Mock T, FcRH5 CAR-T, FcRH5/BCMA TanCAR-T and BCMA/FcRH5 TanCAR-T cells were treated with 5μg/ml recombinant IRTA2a protein or IRTA2c protein for 24 hours, and cell-free supernatants were harvested for evaluating IFN-γ secretion by ELISA. Mean±SD, n=3 biologically independent co-cultures. Experiment was repeated with 2 different donors.

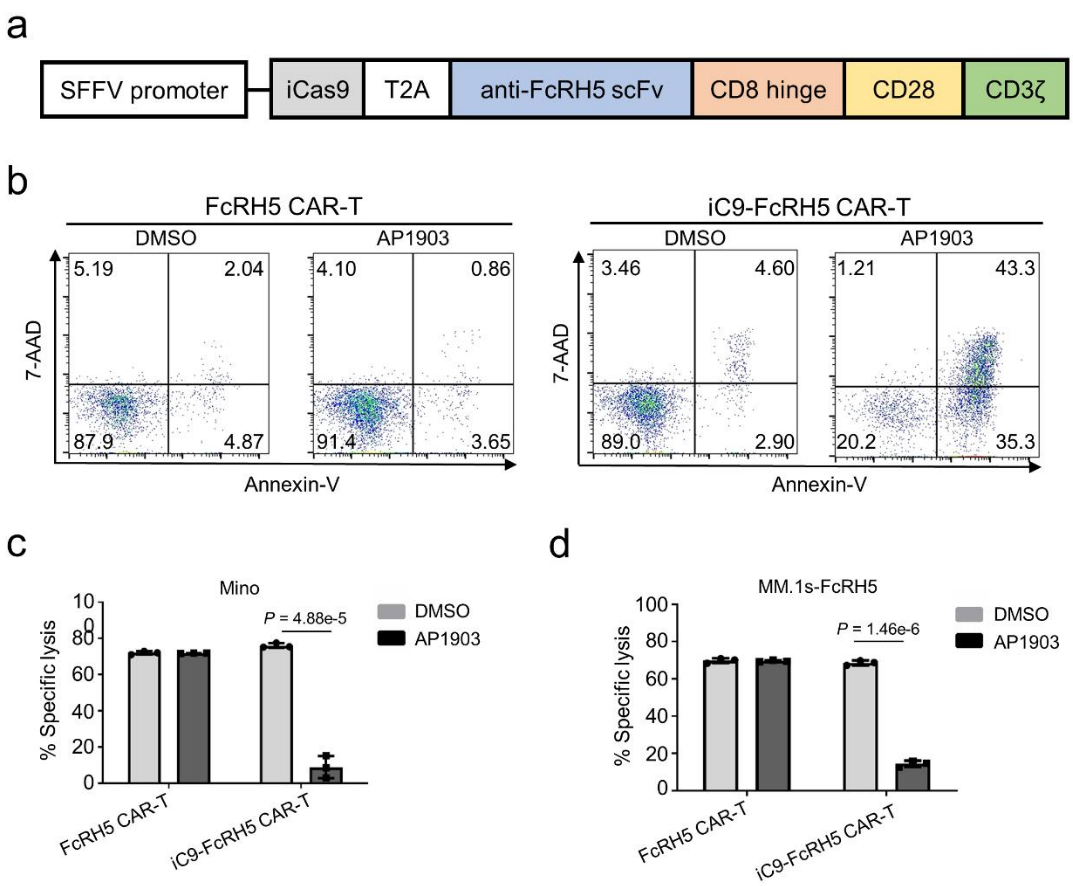


**Supplementary Figure 14. Incorporation of icaspase9 (iC9) suicide gene system improves the safety of FcRH5 CAR-T cells.** (a) Schematic representation of the iC9-FcRH5 CAR expression vector. (b) Human OKT3-activated bulk T cells were transduced with lentiviruses encoding iC9-FcRH5 CAR to generate iC9-FCRH5 CAR-T cells. iC9-FcRH5 CAR-T cells or the control FcRH5 CAR-T cells were treated with 10 nM AP1903 or DMSO for 24 hours, and apoptosis was detected by flow cytometric analysis following Annexin-V/7-AAD staining. Experiment was repeated with 2 different donors. (c,d) iC9-FcRH5 CAR-T or the control FcRH5 CAR-T cells were exposed to 10 nM AP1903 or DMSO for 24 hours, and then Mino cells (c) or MM.1s-FcRH5 cells (d) were added at an E/T ratio of 2:1. After incubation for 6 hours, the luciferase-based cytotoxicity was performed. Treatment with the chemical inducer of dimerization (CID), AP1903 induced rapid and dramatic apoptosis exclusively in iC9-FcRH5 CAR-T cells with a consequent loss of cytolytic activity. Mean±SD, n=3 biologically independent co-cultures, two-tailed Student *t*-test. Experiment was repeated with 2 different donors.

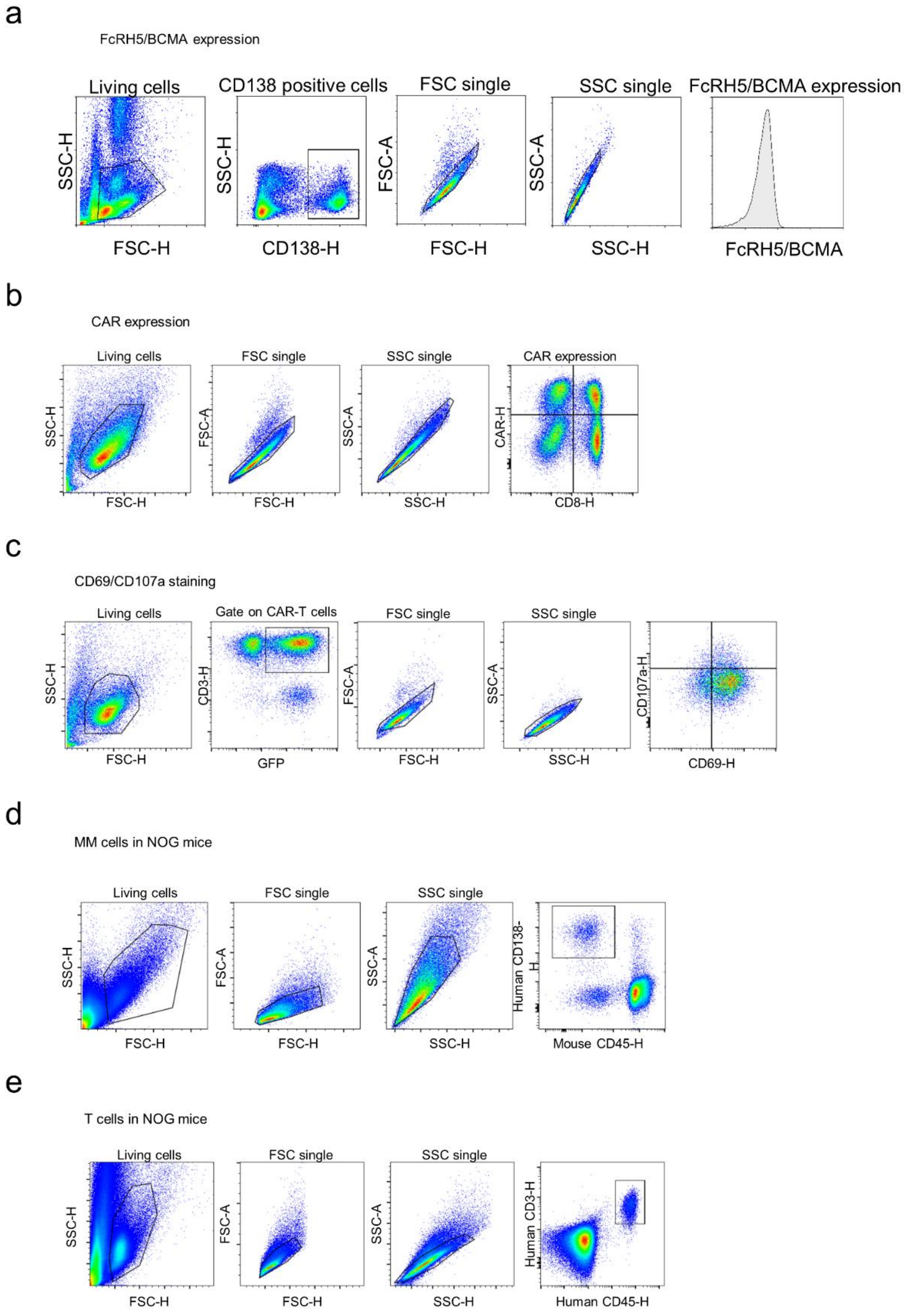


**Supplementary Figure 15. Graphs depicted the gating strategy used in the flow cytometry analysis.** (a) Primary MM cells were isolated from patients and single CD138$^{+}$ cells were gated to analyze BCMA/FcRH5 expression. This gating strategy is applied to the main figure 1a, 1b, 1c, 1d, 1f, 2e, supplementary figure 5b. (b) Staining strategy of CAR expression. Single T cells were

gated to identify CD8 and CAR expression. This gating strategy is applied to the supplementary figure 2a. (c) Single $hCD3^+GFP^+$ CAR-T cells were gated to detect CD69 and CD107a expression after co-cultured with the target cells. This gating strategy is applied to the supplementary figure 3a. (d,e) Spleen, blood and bone marrow cells were collected from NOG mice, single $mCD45^-$ $hCD138^+$ cells were gated to detect MM cells (d), and single human $CD45^+$ $CD3^+$ cells. This gating strategy is applied to the supplementary figure 7e, 7f.

| Oligonucleotides | | |
|---|---|---|
| **sgRNAs primer sequences** | | |
| **Gene name** | **Forward sequence** | **Reverse Sequence** |
| human BCMA | 5’-CACCGGAAGAACATCGAAGTTGACA-3' | 5’-AAACTGTCAACTTCGATGTTCTTCC-3' |
| **Cloning primer sequences** | | |
| **Gene name** | **Forward sequence** | **Reverse Sequence** |
| human FcRH5 | 5’-GGACTAGTATGCTGCTGTGGGTGATATTA-3' | 5’-ATAAGAATGCGGCCGCTCATCTGTGAGGAGCTGAGGA-3' |
| Click beetle red luciferase (CBR) | 5’-TGCTCTAGAATGGTAAAGCGTGAGAAAAAATG-3' | 5’-CCGGAATTCCTAACCGCCGGCCTTCACCAAC-3' |
| Firefly luciferase (FFL) | 5’-TGCTCTAGAATGGAAGACGCCAAAAACATA-3' | 5’-ATAAGAATGCGGCCGCTTACACGGCGATCTTTCCGCC-3' |

**Supplementary table 1. The oligonucleotide sequences of sgRNAs primers and cloning primers.**
