# Supplementary material for: Chimeric antigen receptor T cells targeting FcRH5 provide robust tumour-specific responses in murine xenograft models of multiple myeloma
Source: Nat Commun. 2023 Jun 20;14:3642. doi: 10.1038/s41467-023-39395-4 (PMC10282100; doi:10.1038/s41467-023-39395-4)
Supplement: Supplementary file 3 — Reporting Summary [file 41467_2023_39395_MOESM3_ESM.pdf]

nature portfolio

Corresponding author(s): JIANHONG CHU

Last updated by author(s): May 21, 2023

Reporting Summary

Nature Portfolio wishes to improve the reproducibility of the work that we publish. This form provides structure for consistency and transparency in reporting. For further information on Nature Portfolio policies, see our Editorial Policies and the Editorial Policy Checklist.

Statistics

For all statistical analyses, confirm that the following items are present in the figure legend, table legend, main text, or Methods section.

n/a	Confirmed	
☐	☒	The exact sample size (*n*) for each experimental group/condition, given as a discrete number and unit of measurement
☐	☒	A statement on whether measurements were taken from distinct samples or whether the same sample was measured repeatedly
☐	☒	The statistical test(s) used AND whether they are one- or two-sided<br>*Only common tests should be described solely by name; describe more complex techniques in the Methods section.*
☒	☐	A description of all covariates tested
☐	☒	A description of any assumptions or corrections, such as tests of normality and adjustment for multiple comparisons
☐	☒	A full description of the statistical parameters including central tendency (e.g. means) or other basic estimates (e.g. regression coefficient) AND variation (e.g. standard deviation) or associated estimates of uncertainty (e.g. confidence intervals)
☐	☒	For null hypothesis testing, the test statistic (e.g. F, t, r) with confidence intervals, effect sizes, degrees of freedom and P value noted<br>*Give P values as exact values whenever suitable.*
☒	☐	For Bayesian analysis, information on the choice of priors and Markov chain Monte Carlo settings
☒	☐	For hierarchical and complex designs, identification of the appropriate level for tests and full reporting of outcomes
☒	☐	Estimates of effect sizes (e.g. Cohen's *d*, Pearson's *r*), indicating how they were calculated

Our web collection on statistics for biologists contains articles on many of the points above.

Software and code

Policy information about availability of computer code

Data collection	1. Flow cytometry data was collected on NovoCyte flow cytometer using NovoExpress v1.5.0 software.<br>2. Bioluminescence imaging of mice was acquired with IVIS Lumina III Series System (PerkinElmer)
Data analysis	1. Flow cytometry data was analyzed using FLowjo v10.6.2.<br>2. Graphpad Prism v8 was used for figure generation and statistical analyses.

For manuscripts utilizing custom algorithms or software that are central to the research but not yet described in published literature, software must be made available to editors and reviewers. We strongly encourage code deposition in a community repository (e.g. GitHub). See the Nature Portfolio guidelines for submitting code & software for further information.

Data

Policy information about availability of data

All manuscripts must include a data availability statement. This statement should provide the following information, where applicable:

- Accession codes, unique identifiers, or web links for publicly available datasets
- A description of any restrictions on data availability
- For clinical datasets or third party data, please ensure that the statement adheres to our policy

Analysis of FcRH5, CD19 or BCMA transcript expression in human normal tissue or cells was based on publically avaliable data from The Human Protein Atlas. Web links used in our study: FcRH5 (https://www.proteinatlas.org/ENSG00000143297-FCRL5/tissue); FcRH5 (https://www.proteinatlas.org/ENSG00000143297-FCRL5/

single+cell+type); CD19 (https://www.proteinatlas.org/ENSG00000177455-CD19/single+cell+type); BCMA (https://www.proteinatlas.org/ENSG00000048462-TNFRSF17/single+cell+type). All our data supporting the findings of this study are available within the article and its Supplementary Information as well as Supplementary Data files. Source data are provided as a Source Date file. Source data are provided with this paper.

Human research participants

Policy information about studies involving human research participants and Sex and Gender in Research.

Reporting on sex and gender	The blood or bone marrow samples were collected from myeloma patients or normal donors. The information about the age and sex (or gender) was not collected.
Population characteristics	Healthy donors and multiple myeloma patients. The information about the age and sex (or gender) was not collected.
Recruitment	The peripheral blood or bone marrow samples from healthy donors or multiple myeloma patients were provided by Hematological BioBank. Informed consent was obtained from all healthy donors or patients for use of their samples for laboratory research purposes. No self-selection bias that may impact the results was expected since the samples collected were only used for the in vitro analysis or for the animal studies.
Ethics oversight	The study was approved by the Faculty Hospital Ethics Committee at the First Affiliated Hospital of Soochow University. Because the specimens or data were not collected specifically for our study and no one of our study team has access to the subject identifiers linked to the specimens or data, our study may be not considered as human subjects research.

Note that full information on the approval of the study protocol must also be provided in the manuscript.

Field-specific reporting

Please select the one below that is the best fit for your research. If you are not sure, read the appropriate sections before making your selection.

[x] Life sciences   [ ] Behavioural & social sciences   [ ] Ecological, evolutionary & environmental sciences

For a reference copy of the document with all sections, see nature.com/documents/nr-reporting-summary-flat.pdf

Life sciences study design

All studies must disclose on these points even when the disclosure is negative.

Sample size	No statistical methods were used to predetermine the sample size. Sample size were estimated based on our previous studies testing the anti-tumor effect of Igbeta-specific CAR-T cells (Leukemia. 2020;34(3):821-830.) as well as CS1-specific CAR-T cells (Clin Cancer Res. 2014 ;20 (15):3989-4000.), with an effort to achieve a minimum of n=5 mice per treatment group which proved to be sufficient to reproducibly observe a statistically significant difference.
Data exclusions	No data was excluded from analysis.
Replication	The in vitro and in vivo experiments were repeated in replicates and/or from different subjects in independent experiments. In vitro studies were performed at least three times with similar results. In vivo studies were performed using CAR-T cells from 2 or 3 different donors and the similar results were obtained. Because of the size and expense of the in vivo experiments, they were carried out once.
Randomization	For in vivo tumor models, mice were randomized prior to CAR-T treatment to ensure equivalent average base line tumor burden among groups. For in vitro studies, samples were randomly divided into different experimental groups.
Blinding	For the in vitro experiments, blinding was not performed, however, the authors agree that samples were processed uniformly when acquiring data regardless of whether they were controls or experimental samples. In addition, since the results of our in vitro studies are primarily quantitative rather than subjective evaluation, blinding was not necessary. For in vivo tumor measurement, investigators were blinded during the experiment.

Reporting for specific materials, systems and methods

We require information from authors about some types of materials, experimental systems and methods used in many studies. Here, indicate whether each material, system or method listed is relevant to your study. If you are not sure if a list item applies to your research, read the appropriate section before selecting a response.

Materials & experimental systems

n/a	Involved in the study	
☐	☒	Antibodies
☐	☒	Eukaryotic cell lines
☒	☐	Palaeontology and archaeology
☐	☒	Animals and other organisms
☒	☐	Clinical data
☒	☐	Dual use research of concern

Methods

n/a	Involved in the study	
☒	☐	ChIP-seq
☐	☒	Flow cytometry
☒	☐	MRI-based neuroimaging

Antibodies

Antibodies used

1. Antibodies for Western blotting:
CD3ζ (Santa Cruz, catalog number:sc-166435, clone:E3), GAPDH (Biolegend, catalog number:649203, clone:FF26A/F9), horseradish peroxidase–conjugated goat anti-mouse IgG antibody (Santa Cruz, catalog number:sc-516102)
2. Antibodies for flow ctyometry:
CD138-PE (BD Bioscience, catalog number:552026, clone:MI15)
BCMA-APC (Biolegend, catalog number:357505, clone:19F2)
CD307e(FcRH5)-APC (Biolegend, catalog number:340306, clone:509f6)
CD3-APC (Biolegend, catalog number:300412, clone:UCHT1)
CD3-BV421 (BD Bioscience, catalog number:563798, clone:UCHT1)
Ki-67-APC (Biolegend, catalog number:350514, clone:Ki-67)
allophycocyanin (APC)-conjugated streptavidin (Jackson ImmunoResearch, catalog number:016-130-084)
CD69-BV421 (BD Bioscience, catalog number:562884, clone:FN50)
CD107a-APC (BD Bioscience, catalog number:560664, clone:H4A3)
APC Mouse IgG1 κ Isotype Control(BD Bioscience, catalog number:554681, clone:H4A3)
GranzymeB-Alexa Fluor647 (BD Bioscience, catalog number:560212, clone:GB11)
CD19-APC (Biolegend, catalog number:363006, clone:SJ25C1)
CD56-PE-Cy7 (BD Bioscience, catalog number:557747, clone:B159)
FITC anti-human CD14 Antibody (Biolegend, catalog number:301804, clone:M5E2)
CD34-APC (Miltenyi Biotec, catalog number:130-098-139, clone:AC136)
CD8-Pacific Blue (Biolegend, catalog number:344718, colne:RPA-T8)
Annexin V-APC (Biolegend, catalog number:640920)
7-AAD (BD Bioscience, catalog number:559925)
biotin-labeled goat anti-mouse IgG F(ab')2 polyclonal antibody (Jackson ImmunoResearch, catalog number :115-066-072)
normal polyclonal goat IgG antibody (Jackson ImmunoResearch, catalog number:005-060-003)
3. Antibodies for T cell activation:
ultra-LEAFTM purified anti-Human CD3 antibody (Biolegend, catalog number: 317326, clone:OKT3)
ultra-LEAFTM purified anti-Human CD28 antibody (Biolegend, catalog number 302934, clone:CD28.2)

Validation

All the antibodies were validated for use in flow cytometry. Data was available on the manufacturer's website. All the antibodies used were commercially available.
1. Flow cytometry analysis
CD138-PE (BD Bioscience, catalog number:552026, clone:MI15):
https://www.bdbiosciences.com/zh-cn/products/reagents/flow-cytometry-reagents/research-reagents/single-color-antibodies-ruo/pe-mouse-anti-human-cd138.552026
BCMA-APC (Biolegend, catalog number:357505, clone:19F2): https://www.biolegend.com/en-us/products/apc-anti-human-cd269-bcma-antibody-9115
CD307e(FcRH5)-APC (Biolegend, catalog number:340306, clone:509f6):
https://www.biolegend.com/en-us/products/apc-anti-human-cd307e-fcrl5-antibody-10388
CD3-APC (Biolegend, catalog number:300412, clone:UCHT1):
https://www.biolegend.com/en-us/products/apc-anti-human-cd3-antibody-861
CD3-BV421 (BD Bioscience, catalog number:563798, clone:UCHT1):
https://www.bdbiosciences.com/en-us/products/reagents/flow-cytometry-reagents/research-reagents/single-color-antibodies-ruo/bv421-mouse-anti-human-cd3.563798
Ki-67-APC (Biolegend, catalog number:350514, clone:Ki-67):
https://www.biolegend.com/en-us/products/apc-anti-human-ki-67-antibody-7531
allophycocyanin (APC)-conjugated streptavidin (Jackson ImmunoResearch, catalog number:016-130-084)
https://www.jacksonimmuno.com/catalog/products/016-130-084
CD69-BV421 (BD Bioscience, catalog number:562884, clone:FN50): https://www.bdbiosciences.com/en-us/products/reagents/flow-cytometry-reagents/research-reagents/single-color-antibodies-ruo/bv421-mouse-anti-human-cd69.562884
CD107a-APC (BD Bioscience, catalog number:560664, clone:H4A3):
https://www.bdbiosciences.com/en-us/products/reagents/flow-cytometry-reagents/research-reagents/single-color-antibodies-ruo/apc-mouse-anti-human-cd107a.560664
APC Mouse IgG1 κ Isotype Control (BD Bioscience, catalog number:554681, clone:H4A3): https://www.bdbiosciences.com/en-us/products/reagents/flow-cytometry-reagents/research-reagents/flow-cytometry-controls-and-lysates/apc-mouse-igg1-isotype-

control.554681
Granzyme B-Alexa Fluor647 (BD Bioscience, catalog number:560212, clone:GB11):
https://www.bdbiosciences.com/en-us/products/reagents/flow-cytometry-reagents/research-reagents/single-color-antibodies-ruo/alexa-fluor-647-mouse-anti-human-granzyme-b.560212
CD19-APC (Biolegend, catalog number:363006, clone:SJ25C1):
https://www.biolegend.com/en-us/products/apc-anti-human-cd19-antibody-10264
CD56-PE-Cy7 (BD Bioscience, catalog number:557747, clone:B159):
https://www.bdbiosciences.com/en-us/products/reagents/flow-cytometry-reagents/research-reagents/single-color-antibodies-ruo/pe-cy-7-mouse-anti-human-cd56-ncam-1.557747
CD34-APC (Miltenyi Biotec, catalog number:130-098-139, clone: AC136): https://www.miltenyibiotec.com/US-en/products/cd34-antibody-anti-human-ac136.html#conjugate=apc:size=100-tests-in-200-ul
CD8-Pacific Blue (Biolegend, catalog number:344718, colne:RPA-T8):
https://www.biolegend.com/en-us/products/pacific-blue-anti-human-cd8-antibody-6509
Annexin V-APC (Biolegend, catalog number:640920): https://www.biolegend.com/en-us/products/apc-annexin-v-8144
7-AAD (BD Bioscience, catalog number:559925): https://www.biolegend.com/en-us/products/apc-annexin-v-8144
biotin-labeled goat anti-mouse IgG F(ab')2 polyclonal antibody (Jackson ImmunoResearch, catalog number :115-066-072)
https://www.jacksonimmuno.com/catalog/products/115-066-072
normal polyclonal goat IgG antibody (Jackson ImmunoResearch, catalog number:005-060-003)
https://www.jacksonimmuno.com/catalog/products/005-060-003
2. Western blot
CD3ζ (Santa Cruz, catalog number:sc-166435, clone:E-3):
https://www.scbt.com/zh/p/cd3-zeta-antibody-e-3
GAPDH (Biolegend, catalog number:649203, clone:FF26A/F9):
https://www.biolegend.com/en-us/products/direct-blot-hrp-anti-gapdh-antibody-12771
horseradish peroxidase–conjugated goat anti-mouse IgG antibody (Santa Cruz, catalog number:sc-516102):
https://www.scbt.com/p/m-igg-kappa-bp-hrp?requestFrom=search
3. T cell activation
ultra-LEAFTM purified anti-Human CD3 antibody (Biolegend, catalog number:317326, clone:OKT3)
https://www.biolegend.com/en-us/products/ultra-leaf-purified-anti-human-cd3-antibody-7745
ultra-LEAFTM purified anti-Human CD28 antibody (Biolegend, catalog number:302934, clone:CD28.2)
https://www.biolegend.com/en-us/products/ultra-leaf-purified-anti-human-cd28-antibody-7743

Eukaryotic cell lines

Policy information about cell lines and Sex and Gender in Research

Cell line source(s)	NCI-H929, MINO, MM.1s and HEK-293T cells were obtained from American Type culture Collection (ATCC).
Authentication	Cell lines were routinely tested for expression of essential or typical cell surface markers, no other authentication was performed.
Mycoplasma contamination	All cell lines tested were negative for mycoplasma contamination.
Commonly misidentified lines (See ICLAC register)	No commonly misidentified cell lines were used in this study.

Animals and other research organisms

Policy information about studies involving animals; ARRIVE guidelines recommended for reporting animal research, and Sex and Gender in Research

Laboratory animals	Male NOD.Cg-PrkdcscidIl2rgtm1Sug/JicCrl (NOG) mice aged 6-8 weeks were purchased from Vital River Laboratories (Beijing, China) and maintained at the Animal Facility of Soochow University under constant environmental conditions with a 12h light/dark cycle and a temperature range of 20-22℃ and the relative humidity of 40-70%.
Wild animals	No wild animals were used.
Reporting on sex	The sex of the mice was not considered in the study design and analysis, and only male mice were used in the tumor models to ensure gender uniformity.
Field-collected samples	No filed-collected samples were used.
Ethics oversight	All mouse experiments were performed with ethical compliance and approved by the committee of Animal care and Use of Soochow University.

Note that full information on the approval of the study protocol must also be provided in the manuscript.

Flow Cytometry

Plots

Confirm that:

- [x] The axis labels state the marker and fluorochrome used (e.g. CD4-FITC).
- [x] The axis scales are clearly visible. Include numbers along axes only for bottom left plot of group (a 'group' is an analysis of identical markers).
- [x] All plots are contour plots with outliers or pseudocolor plots.
- [x] A numerical value for number of cells or percentage (with statistics) is provided.

Methodology

Sample preparation	For surface staining, cells were incubated with antibodies on ice for 20 minutes. For intracellular staining, cells were fixed and permeabilized using Cytofix /Cytoperm (BD Biosciences) for 30 minutes at room temperature and washed with 1XPermWash (BD Biosciences). Subsequent staining was performed using 1xPerwash as staining and wash buffer.
Instrument	ACEA Novocyte 5150 flow cytometer
Software	NovoExpress v1.5.0; Flowjo v10.6.2
Cell population abundance	Post-sort purity was always assessed and confirmed prior to use in the experiment.
Gating strategy	Generally, cells were firstly gated by FSC-H/SSC-H to exclude debris including dead cells, and then FSC-A/FSC-H and SSC-A/SSC-H was used to exclude doublets.

- [x] Tick this box to confirm that a figure exemplifying the gating strategy is provided in the Supplementary Information.
